# The Administration of Charity

**Published:** 1907-10-05

**Authors:** 


### THE ADMINISTRATION OF CHARITY.

Under this heading, the *Times* publishes one article occupying two columns, which would appear to be the first of a series. As it stands it is complete in itself, and seeing that it appeared a week ago, and no second has followed, the impression may be due to a mere printer's error. It is evidently from the pen of one of the older sympathisers with charity organisation methods, and it is surprising, having regard to the developments which have taken place in the last fifteen years, that the first two-thirds of it should consist mainly of views which hardly apply to the better charities of to-day. They are too narrow in their conception and ill-adapted to charitable administration of the best kind in these modern days. Further, the article is remarkably wanting in facts, being very thin in this respect, and the instances quoted are anything but convincing to those who are familiar with the field of charity and have had many years' experience covering every branch of the work. So far as he has gone, the only clear note which the writer has struck is that he wants to secure some central controlling body, an idea which the Charity Organisation Society has kept constantly before the public for many decades, and which, so far as practical politics are concerned, is probably further divorced from fruition than ever before in the history of the voluntary institutions of the United Kingdom.

If we attempt to consider the reason for this failure to treat a great subject in an illuminating and modern spirit, it is not far to seek. Mr. C. S. Loch, the veteran economist and organiser, has stuck to his guns and done yeoman service in reducing to order and productive cultivation many portions of the field of charity which would otherwise have yielded little or nothing but tares. With him, as with many others, *anno domini* is making progress, and there is no one connected with the Charity Organisation Society to take his place or carry on his work. Whether it be charity organisation or the administration of the affairs of the smallest individual charity, the pressing need of the moment is identical, and until it is met there is little hope of any real and wise solution of the problems which underlie the reduction of waste, the stopping of expenditure by obsolete charities or institutions which have fulfilled their mission, the staying of wasteful expenditure without adequate result, and the absence of business methods in the expenditure of eleemosynary funds. This pressing need is an army of men and women workers who, as the writer in the *Times* wisely insists, should be found to a large extent amongst the subscribers and supporters of individual charities, with whom rests the responsibility for the present state of affairs. It is as true to-day, as ever it was in the history of the world, that he who pays the piper can command the tune. That these workers exist, and that they will give adequate time and invaluable service in a good cause, has been demonstrated in recent years by the establishment of university settlements in the poorer districts of great cities, and by the gratifying success of a movement like that of the League of Mercy, which now numbers between two and three hundred thousand workers on its books. Of these workers, probably twenty thousand give deliberately and willingly a considerable measure of time each year to personal service in the cause of the sick in the days of health.

Many attempts in many directions, and in almost every section of the field of charity, have been made from time to time to secure the co-operation of the various agencies with kindred objects, so as to prevent overlapping and to provide that the huge revenue of ten millions sterling, which is available for charitable purposes in London alone at the present time, shall be made to yield the maximum of result in benefit not only to the poor, the needy, and the sick, but to the whole community and the state. With the omission of a very few and imperfect exceptions co-operation has everywhere failed to flourish. The reason is that almost every institution has its own circle of warm supporters, who regard it as their special privilege to mismanage its affairs, and to exclude others from participation in the work, which otherwise might often be materially improved. Again, when an attempt is made to secure co-operation amongst various workers connected with churches and religious bodies, any experienced mind which may devote itself to a crusade of the kind is speedily made aware that, at the bottom, no church or religious body really desires co-operation. The reason for this springs from a feeling of jealousy, which is often unconsciously held, and from the further and more important belief felt in their hearts.

by the leaders of most churches. This belief is that if the various religious bodies, through their leaders, were to be sunk in one great council, which collectively could make the moneys available go fifty to one hundred per cent. further than under the individual system, the other fellow would somehow or other get the benefit, and that individual churches must consequently lose something of what they already possess. In any case they would suffer interference and possibly loss of prestige, from which they are entirely free, as they believe, when they stand apart and mess the business of charity to their heart's content.

We have said that the key to the solution of the prevention of the waste of charitable funds in London to-day lies with the subscribers who supply the money. In the same way the introduction of co-operation, resulting in the complete overtaking of the work of spiritualising the whole of the inhabitants of a great city like the metropolis of the Empire, must at the bottom rest, with the individual members of each congregation and church who, did they but feel, as men of business and thoughtful individual forces, that better methods should prevail, might bring about complete co-operation within a few months of the moment, when the spirit touched the hearts of the people and stirred them up to secure this essential result. If it were possible to move the minds of the masses of the population, to passionately desire nothing but the highest and best organisation and work at the hands of the churches and charities of the metropolis, what a miracle might speedily be wrought in everything which affects the spiritual life and social welfare of the people of London.

